# Experimental Motor Neuron Disease Induced in Mice with Long-Term Repeated Intraperitoneal Injections of Serum from ALS Patients

**DOI:** 10.3390/ijms20102573

**Published:** 2019-05-25

**Authors:** Izabella Obál, Bernát Nógrádi, Valéria Meszlényi, Roland Patai, Gerda Ricken, Gabor G. Kovacs, Kornélia Tripolszki, Márta Széll, László Siklós, József I. Engelhardt

**Affiliations:** 1 Department of Neurology, University of Szeged, H-6725 Szeged, Hungary; obalizabella@yahoo.com (I.O.); bernatnogradi@gmail.com (B.N.); mesval13@gmail.com (V.M.); 2 Department of Neurology, Aalborg University Hospital, DK-9000 Aalborg, Denmark; 3 Foundation for the Future of Biomedical Sciences in Szeged, Szeged Scientist Academy, H-6721 Szeged, Hungary; 4 Institute of Biophysics, Biological Research Centre of the Hungarian Academy of Sciences, H-6726 Szeged, Hungary; roland.patai.1988@gmail.com (R.P.); siklos.laszlo@brc.mta.hu (L.S.); 5 Clinical Institute of Neurology, Medical University of Vienna, Vienna A-1090, Austria; gerda.ricken@meduniwien.ac.at (G.R.); gabor.kovacs@meduniwien.ac.at (G.G.K.); 6 Department of Medical Genetics, University of Szeged, H-6720 Szeged, Hungary; tripolszki.kornelia@med.u-szeged.hu (K.T.); szell.marta@med.u-szeged.hu (M.S.); 7 Hungarian Academy of Sciences – University of Szeged, Dermatological Research Group, H-6720 Szeged, Hungary

**Keywords:** ALS, motor neuron, degeneration, serum, passive transfer, IgG uptake, intracellular calcium

## Abstract

In an earlier study, signs of commencing degeneration of spinal motor neurons were induced in mice with short-term intraperitoneal injections of immunoglobulin G (IgG) taken from patients with amyotrophic lateral sclerosis (ALS). Since in that study, neither weakness nor loss of motor neurons was noted, to test whether the ALS IgG in this paradigm has the potential to evoke relentless degeneration of motor neurons, treatment with repeated injections over a longer period was carried out. Mice were systematically injected intraperitoneally with serum taken from ALS patients over a 75-day period. At selected time points, the isometric force of the limbs, number of spinal motor neurons and their intracellular calcium levels were determined. Furthermore, markers of glial activation and the motoneuronal uptake of human IgG were monitored. During this period, gliosis and progressive motoneuronal degeneration developed, which led to gradual loss of spinal motor neurons, more than 40% at day 21, along with decreasing muscle strength in the limbs. The inclusion-like accumulation of IgG appeared in the perikarya with the increase of intracellular calcium in the cell bodies and motor nerve terminals. Our results demonstrate that ALS serum can transfer motor neuron disease to mice.

## 1. Introduction

Amyotrophic lateral sclerosis (ALS) is a rare but fatal neurodegenerative disease involving upper (cortical) and lower (brain stem and spinal cord) motor neurons (MNs). The consequence of the loss of lower MNs is severe striated muscle atrophy with paralysis, which eventually leads to death, principally by respiratory failure [1]. The disease is incurable and its causes are still obscure, but some main components of the pathomechanism of MN degeneration have been proposed [2]. Here, we will focus on the oxidative stress [3], excitotoxicity [4], mitochondrial dysfunction [5] and genetic alterations. Ten percent of the patients had the familiar form of the disease and 90 percent appeared to be sporadic. However, genetic alterations were found in both groups. More than 35 major ALS related genes, about 99 ALS risk genes and about 160 alterations of the genes of other neurological diseases were identified in ALS patients. Some genetic alterations may also be the targets of future gene therapies [6]. However, all of these theories lack the explanation of the selective injury of MNs.

In our pioneering studies, we proved that selective abnormalities of motor neurons can be induced experimentally by immunizing guinea pigs and goats with purified bovine spinal motor neurons or the homogenate of the ventral horn of the spinal cords [7]. Some of the abnormalities could be transferred with isolated immunoglobulin G (IgG) from these animals to mice via intraperitoneal (ip) injections. These were increased acetylcholine release from the axon terminals of spinal motor neurons [8], IgG accumulation and increased intracellular calcium in these motor neurons [9]. We intended to use these data values as a guide for exploring the putative autoimmune background of ALS. In ALS patients, similar pathological changes were detected as IgG accumulated in the motor neurons [10] and increased calcium was present in the motor axon terminals [11]. These pathological features, i.e., increased calcium in the motor axon terminals [12] and IgG uptake [13], may also be induced in mice by means of ip inoculation with IgG from ALS patients. The sites of accumulation of IgG were the axon terminals, microtubules, the rough endoplasmic reticulum, the Golgi system and lysosomes [13]). Furthermore, lymphocytic infiltrations [14]), activated microglia [15], an increase in macrophage chemoattractant protein-1 (MCP-1) [16] and tumor necrosis factor alpha (TNF-α) were demonstrated in the spinal cord of deceased patients and in mice injected with IgG from ALS patients and from immunized animals [17]. These data could be interpreted as signs of an immune/inflammatory process which was initiated by the uptake of anti-motoneuronal IgG in the motor neurons. The induced immune/inflammatory reaction may be responsible for the development of the degeneration of motor neurons, both in the animal models and in the human disease.

These findings justified the initiation of screening the sera obtained from ALS patients for autoantibodies using a large-scale (9480 proteins) microarray method [18]. A panel of highly immunoreactive IgG antibodies, specific for ALS, were identified which were directed against 20 human proteins. Most of the proteins are localized to the same ultrastructural organelles in which IgG is accumulated in vivo, to those which were damaged in several genetic forms of ALS and in which increased intracellular calcium is accumulated.

Based on our previous results, supported by this recent evidence of the importance of immune factors in the pathomechanism of ALS, an experimental paradigm was designed to test whether progressively developing motor neuron disease-like symptoms could be induced in mice with a long series of injections of sera taken from ALS patients. For these experiments, instead of IgG, the serum was injected because it has more complex and stronger biological effects than the isolated and partially inactivated IgG after the process of purification. During an 82-day period, mice were regularly injected ip with 0.3 mL serum on 35 occasions at most. Three different surviving groups (day 4, 21 and 82) were created to monitor the development of potential histological alterations, ultrastructural changes and the calcium level of motor neurons. The muscle strength of the animals was tested at roughly evenly-spaced time points throughout the actual survival time of each animal. The evidence of uptake and retrograde transport of IgG antibodies directed to motor neuron components by the motor neurons of the central nervous system was given in the 1980s, but it was not connected to the pathomechanism of human diseases [19,20,21].

## 2. Results

**Figure 1:**
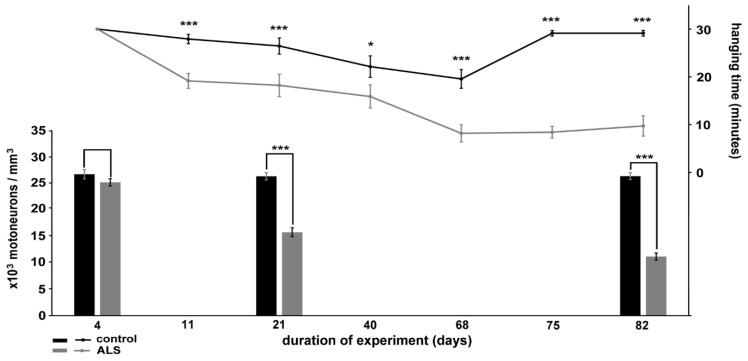
Correlation of diminishing isometric muscle strength (line chart) with the decreasing number of lumbar motor neurons (column graphs) after inoculation with sera taken from control (black) and ALS (grey) patients; data values are shown on the right vertical axis. After a temporary decrease in muscle strength in mice injected with control serum, they regained their strength by the 75th day of the treatment. The muscle strength of mice injected with ALS serum progressively decreased, expressed by the inability to hang on for long, and differed significantly from control mice by day 11 of the treatment. In parallel with the development of the functional deficit in mice injected with ALS serum, the number of motor neurons in the lumbar region of their spinal cords also gradually decreased, and resulted in more than a 50% loss by the end of the treatment. (data values are shown on the left vertical axis; * *p* < 0.05, *** *p* < 0.001, Student t-test; error bars denote the s.e.m.).

The muscle strength of the animals was tested throughout the study, and it was expressed as the maximum time of holding on to some bars while hanging upside down from the bars of the cages (see the video Supplementary Materials and Appendix A). Compared to the control animals, the ALS-serum injected mice were not able to maintain their grasps as long already as early as day 11 of the study (19.16 ± 1.56 min versus 27.92 ± 0.97 min; Figure 1, line chart). At subsequent time points further increasing weakness was observed in the ALS serum-injected mice: day 18: 18.22 ± 2.33 min versus 26.45 ± 1.68 min, day 40: 15.89 ± 2.41 min versus 22.40 ± 2.24, day 68: 8.18 ± 1.81 min versus 19.57 ± 1.96 min, day 75: 8.72 ± 1.22 min versus 29.14 ± 0.59 min, day 80: 9.73 ± 2.12 min versus 29.14 ± 0.59 min. The mice injected with normal serum also exhibited a slight, transient decline in their strength, but they started to recover one week before the discontinuation of the injections (day 68). They completely recovered by the end of injection period (day 75) and remained fit until the end of the experiment (day 82) (Figure 1, line chart).

To reduce the number of animals that participated in the study, the number of motor neurons on day 4, 21 and 82 of the experiment was counted in sections set apart from those prepared for histologic or immunohistochemical characterization. These sections were stained with hematoxylin-eosin. At the first investigated time point, on the fourth day of the experiment, there was no difference in the number of lumbar motor neurons of mice injected intraperitoneally with serum taken from ALS patients (25.05 × 10^3^ / mm^3^ ± 0.69 × 10^3^ / mm^3^) compared to the controls (26.57 × 10^3^ / mm^3^ ± 0.87 × 10^3^ / mm^3^) (Figure 1, column graph). However, on day 21, a significant loss of motor neurons was detected in the lumbar spinal cords of the animals injected with sera taken from ALS patients (15.67 × 10^3^ / mm^3^ ± 0.83 × 10^3^ / mm^3^) compared to the control group (26.19 × 10^3^ / mm^3^ ± 0.69 × 10^3^ / mm^3^) (Figure 1, column graph). In the ALS-serum injected mice a further decrease in the number of motor neurons was seen on day 82 (11.12 × 10^3^ / mm^3^ ± 0.66 × 10^3^ / mm^3^), while there was no loss of motor neurons in the control animals (26.22 × 10^3^ / mm^3^ ± 0.65 × 10^3^ / mm^3^) (Figure 1, column graph).

**Figure 2:**
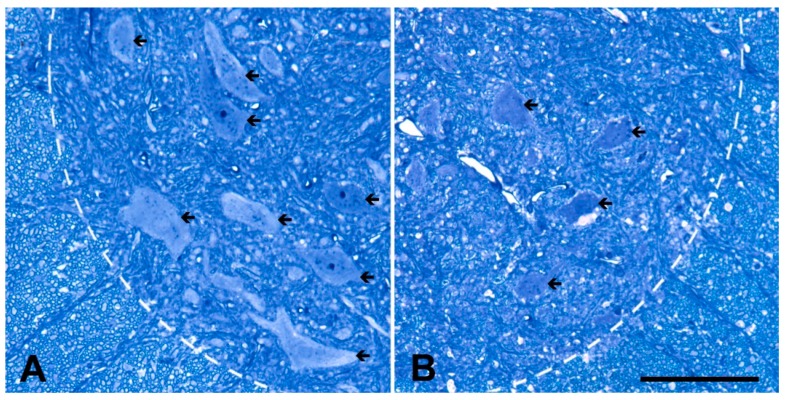
Semithin sections of the ventrolateral view of the spinal cords of mice injected with control (**A**) or ALS (**B**) serum at day 82. Numerous intact motor neuron profiles can be seen in the spinal cord of control serum treated animal (**A**, arrows), while in the spinal cord from mice treated with ALS serum (**B**) only degenerating motor neurons (arrows) can be observed. To indicate the ventrolateral position of the motor neurons, the border of the white matter is marked with dashed lines. Scale bar: 100 µm.

**Figure 3:**
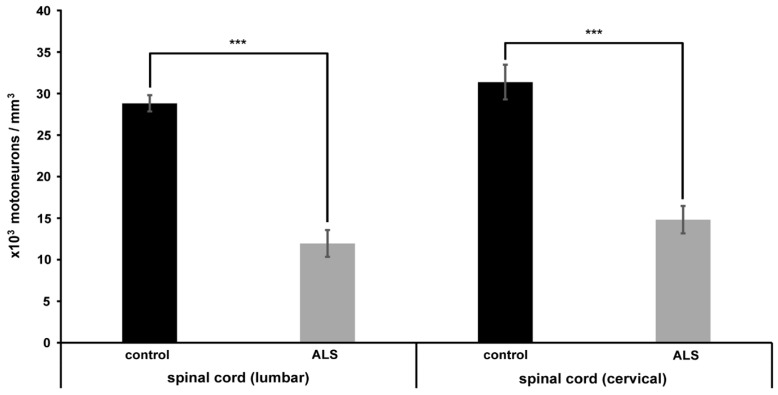
The number of motor neurons in the lumbar and cervical spinal cords of mice injected ip with ALS and control sera. The figure shows the simultaneous loss of motor neurons in the cervical and lumbar regions of the spinal cords of mice injected with ALS serum. The number of motor neurons was determined at the end of the experiment (day 82). A significant (*** *p* < 0.001) and similar loss of motor neurons was documented in these spinal cord regions of mice injected with ALS serum (grey columns) compared to mice injected with control serum (black columns). This suggests that the long-term injections with ALS serum induced degeneration of motor neurons throughout the spinal cords. Here the error bars denote the s.e.m.

At the end of the experiment, the rest of the animals were processed to determine the calcium level in the motor neurons at the cervical and lumbar enlargements of the spinal cord by using an electron microscope. Since the procedure makes it possible to determine the number of motor neurons, a set of semithin plastic sections were also prepared to determine the cell numbers in both the cervical and in the lumbar segments of the spinal cords. Qualitatively, a marked loss of motor neurons can be clearly seen in light microscopic semithin sections (0.5 µm) taken from the lumbar spinal cords (Figure 2). In the sections made from control animals, several large morphologically intact motor neurons were present at the ventrolateral position (Figure 2A). At the same position in a representative section obtained from a mouse treated with ALS serum, only degenerating motor neurons were observed (Figure 2B). To be precise, the number of motor neurons were determined by the physical disector method on day 82. The number of motor neurons in the lumbar spinal cord of the mice injected with ALS serum dropped to 11.95 × 10^3^/ mm^3^ ± 1.63 × 10^3^ / mm^3^ compared to the 28.82 × 10^3^ / mm^3^ ± 0.98 × 10^3^ / mm^3^ of the control serum-injected animals (Figure 3), whose values accord well with those obtained in the histologic sections. In the cervical region, a similar change was documented since the number of motor neurons in ALS-serum treated animals dropped to 14.64 × 10^3^ / mm^3^ ± 1.64 × 10^3^ / mm^3^ compared to the control serum treated group with 31.38 10^3^ / mm^3^ ± 2.09 × 10^3^ / mm^3^ (Figure 3).

**Figure 4:**
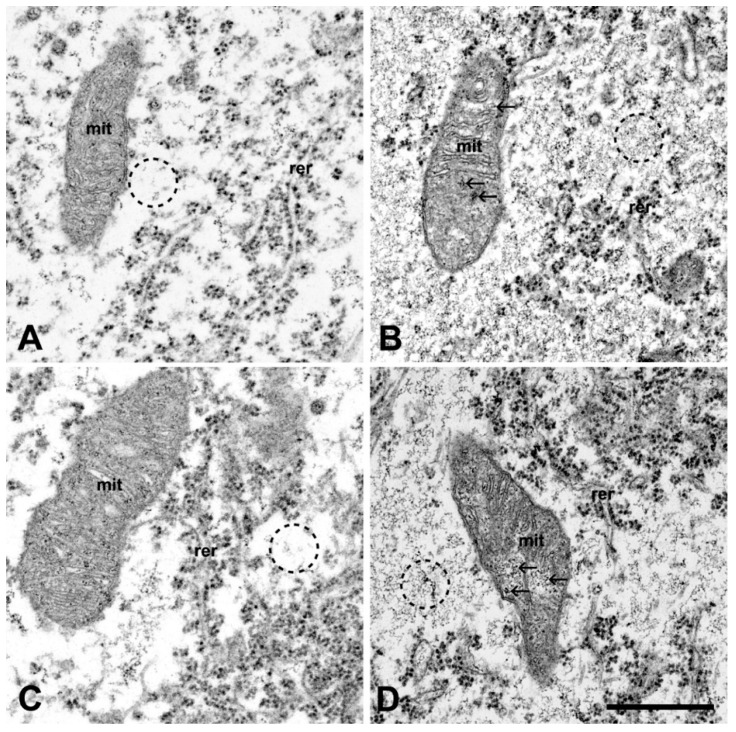
The electron micrographs of the perikaryal area of motor neurons from the cervical (**A**,**B**) and lumbar region (**C**,**D**) of the spinal cords of mice injected with control (**A**,**C**) or ALS-serum (**B**,**D**). The mice at day 82 were fixed with oxalate-pyroantimonate containing solution that traps the calcium in situ, resulting in EDDs. The mitochondria (mit) seemed to be intact in control mice in both spinal cord regions, the rough endoplasmic reticulum (rer) is undamaged with regularly placed ribosomes, and only small clusters of EDDs representing tissue calcium is visible (**A**,**C**). In mice treated with ALS serum, the cristae of the mitochondria (mit) are disorganized and clusters of EDDs are present (arrows). The sacks of the rough endoplasmic reticulum (rer) are dilated, and detached ribosomes can often be seen. In the cytosol, diffuse calcium accumulation (**B**,**D**; encircled) is present as a dark grainy background. There is no such effect on the spinal motor neuron of a mouse inoculated with normal serum. The cytosol is clear (**A**; encircled). Scale bar: 1 µm.

The motor neurons of ALS serum-injected mice exhibited the hallmarks of neuronal degeneration under the electron microscope. Mitochondrial swelling, fragmented and dilated cisterns of the rough endoplasmic reticulum, often with detached ribosomes were noted in the perikarya of the cervical and lumbar motor neurons (Figure 4B,D) compared to controls (Figure 4A,C). The mitochondrial swelling in the perikarya was expressed quantitatively, as well: the relative volume occupied by the mitochondria was 18.38% ± 0.82% in the motor neurons of ALS serum injected mice, which was significantly higher (*p* = 0.033) than the same value in the control mice (15.33% ± 1.10%).

**Figure 5:**
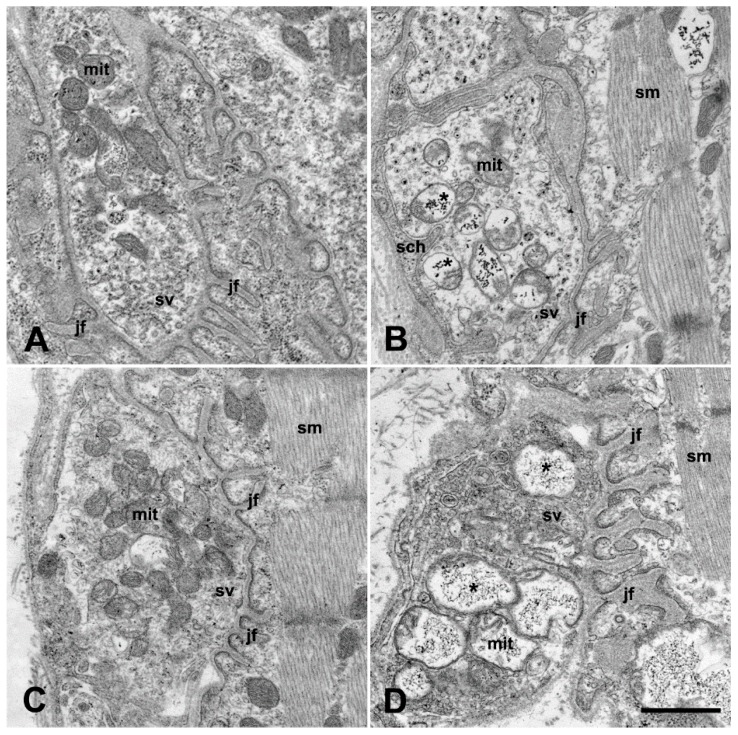
Electron microscopic images of neuromuscular junctions in the fore limb (**A**,**B**) and hind limb (**C**,**D**) from the interosseus muscles of oxalate-pyroantimonate fixed mice (day 82). In the control mice, in both muscles (**A**,**C**), intact mitochondria (mit) are visible within the motor axon terminals. In mice treated with ALS serum (**B**,**D**), swollen mitochondria (mit) with large clusters of EDDs (asterisk) are present in the axon terminals. Irrespective of the treatment, the specialized postsynaptic membrane—the junctional folds (jf)- and the skeletal muscle (sm) seem to be intact in both muscles. Sch: Schwann cell, sv: synaptic vesicles. Scale bar: 1 µm.

**Figure 6:**
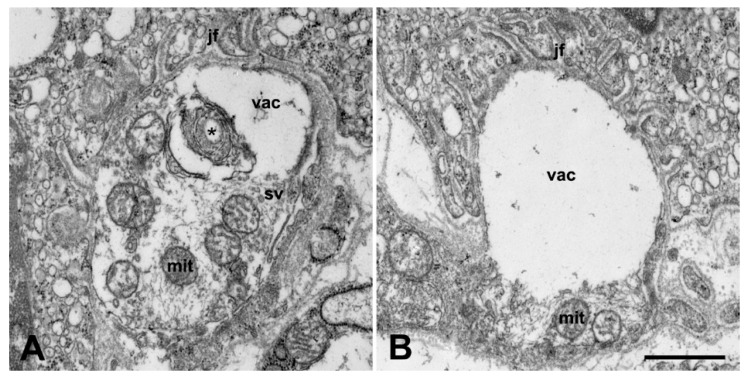
Neuromuscular synapses exhibiting different degrees of degeneration in the interosseus muscle of the hind limb of mice treated with ALS serum (day 82). (**A**): in the motor axon terminal, some mitochondrial profiles (mit) and synaptic vesicles (sv) are still recognizable. A vacuolar degeneration (vac) with debris of unrecognizable structures (asterisk) is visible, which leaves part of the postsynaptic membrane denuded. (**B**): no intracellular organelles are present in a large vacuole (vac) which fills the majority of the space of the presynaptic terminal and results in practically no innervation to the whole surface of the postsynaptic membrane. The presynaptic membrane is lost in this area. jf: junctional folds. Scale bar: 1 µm.

Similar degenerative features were found in the axon terminals of the motor neurons in the interosseous muscles of the fore limb (Figure 5B) and of the hind limb (Figure 5D). Quantitatively, within the axon terminals in the interosseus muscles from ALS serum treated mice, the mitochondrial volume fraction was significantly higher (14.71% ± 0.96%) compared with the volume that was obtained from the control animals (11.55% ± 0.96%) (*p* = 0.0026). Some axon terminals had large vacuoles (Figure 6A), and others were completely destroyed and empty (Figure 6B). In contrast, in the animals inoculated with normal serum there were no signs of degeneration either in the perikarya of the cervical (Figure 4A) or the lumbar motor neurons (Figure 4C) or in the motor axon terminals (Figure 5A,C). Apart from the structural alterations, inoculation with ALS sera evoked a large increase in intracellular calcium, visualized by electron dense deposits (EDDs), both in the perikarya (Figure 4B,D), as well as in the axon terminals (Figure 5B,D) of cervical and lumbar motor neurons.

**Figure 7:**
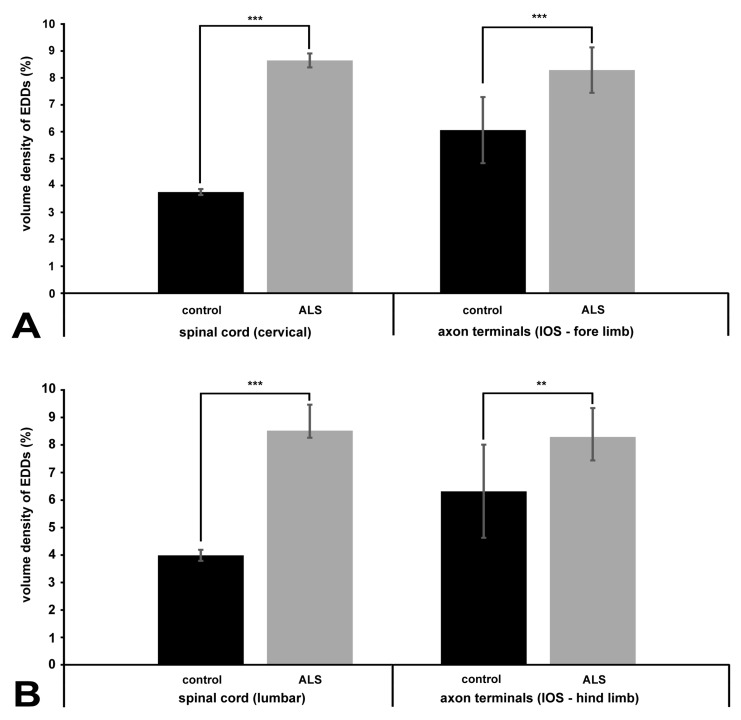
Quantification of the EDDs representing calcium in cervical and lumbar motor neurons and the axon terminals at day 82. (**A**) The perikarya of cervical motor neurons and motor axon terminals in the fore limb interosseus muscles. (**B**) The perikarya of lumbar motor neurons and motor axon terminals in the interosseus muscles of the hind limbs. A significant increase in the amount of EDDs is present in each compartment of ALS serum injected mice (gray columns) compared to the controls (black columns). The data values are expressed as a volume of EDDs relative to the relevant perikaryal and axon terminal reference volumes. ** *p* < 0.01, *** *p* < 0.001, Student t-test. IOS: interosseous muscles. Here, the error bars denote the s.e.m.

The volume fractions of the EDDs were also determined quantitatively. In the perikarya of the cervical spinal cord, the volume fraction of EDDs increased in the ALS serum-treated group (8.65% ± 0.26%) compared to the animals injected with control sera (3.76% ± 0.11%) (Figure 7A). A similar increase was measured in the perikarya of the lumbar motor neurons (8.52% ± 0.94%) compared to the controls (3.99% ± 0.20%) (Figure 7B) The volume fraction of EDDs also increased in the axon terminals of the interosseous muscles both in the fore limbs (Figure 7A) of ALS serum injected mice: 8.29% ± 0.84%) and in the hind limbs (Figure 7B): 8.29% ± 1.85%) compared to the controls in the fore limbs (Figure 7A) 6.06% ± 1.23%) and in the hind limbs: (Figure 7B) 6.32% ± 1.69%)

**Figure 8:**
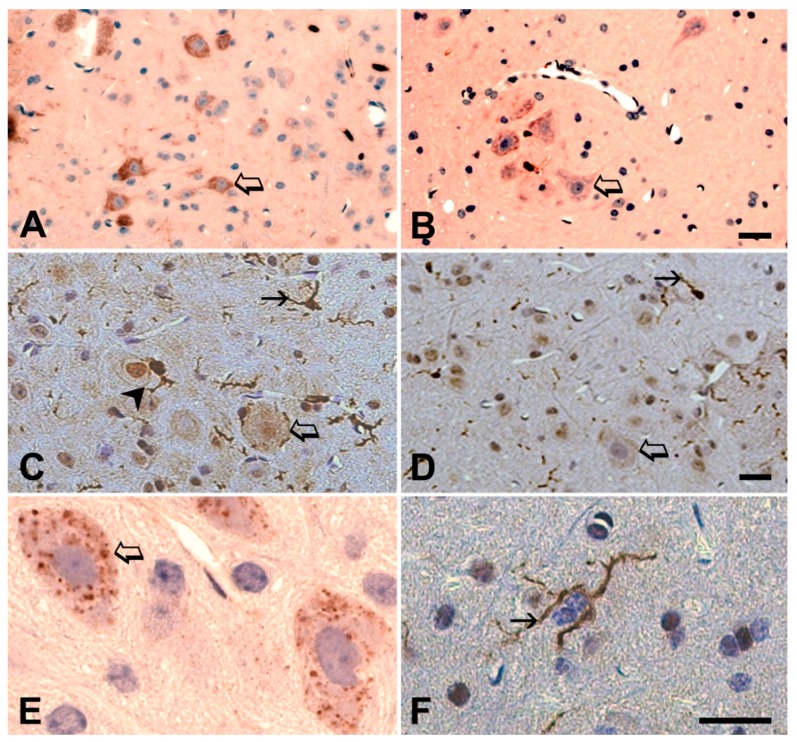
Immunohistochemical detection for IgG (**A**,**B**,**E**) in the motor neurons and for IBA-1 (**C**,**D**,**F**) in the spinal cord of ALS serum-injected and control mice. (**A**): on day 82, the cytoplasm of the motor neurons (hollow arrow) of the mice injected ip with ALS serum are heavily loaded with IgG (dark brown color). (**B**): The spinal motor neurons (hollow arrow) of the animals injected with control serum contain minimal IgG detected by the same method also on day 82 of the experiment. The bar represents 25 µm in figures (**A**,**B**). (**C**): The microglial cells (dark brown color) are enlarged (solid arrow) and their extended processes (arrow head) surround the motor neurons (hollow arrow) in the spinal cord of an animal injected with ALS serum; day 82. (**D**): The processes of the microglia cells in the spinal cord of mice injected with normal serum (day 82) are seen in the parenchyma (solid arrow), but not around the motor neurons (hollow arrow). The cell bodies are smaller (solid arrow) than the microglia of the activated forms in figure (**C**). The bar indicates 35 µm in (**C**,**D**). (**E**): IgG in the ventral horn in the lumbar spinal cord section of a mouse injected with ALS serum on day 21 of treatment, which appears in the cytoplasm of the motor neurons (hollow arrow) and it is particulate and has an inclusion-like appearance. (**F**): On the same day of ALS serum treatment, an activated microglia cells with large processes (solid arrow) are present in the spinal cord. Here the bar scale represents 15 µm in (**E**,**F**).

**Figure 9:**
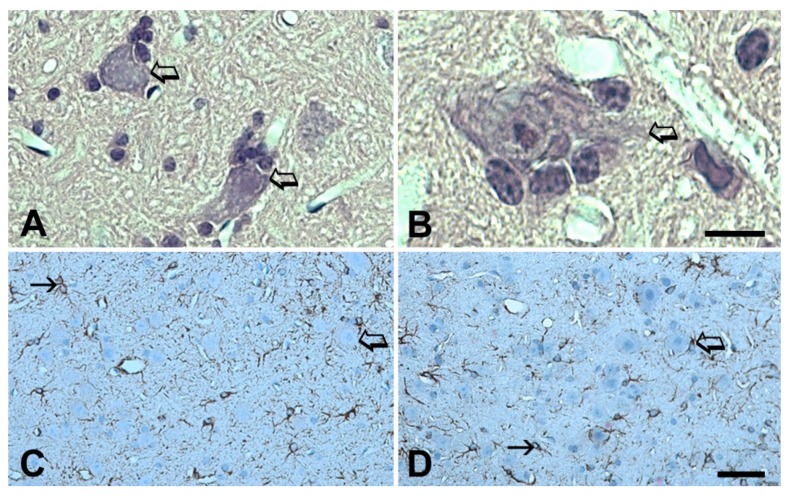
Neuronophagia and astrocytosis in the spinal cord of ALS serum injected mice. Motor neurons (hollow arrows) underwent satellitosis and neuronophagia in the ventral horns of the spinal cord of mice after ip inoculation with ALS sera for 21 days (**A**) and for 82 days (**B**). Hematoxylin-eosin staining; the scale bar represents 10 µm for B and 25 µm for A. Immunostaining for GFAP in the ventral horn of the spinal cord of mice injected ip with sera from ALS patients for 82 days confirms astrocytosis (**C**), while the ventral horn of animals injected with normal human sera (**D**) displays a normal density of astrocytes (indicated by solid arrows). The hollow arrows point to motor neurons. Here the scale bar represents 35 µm for both photographic images.

The histological examinations of the spinal cords on days 4, 21 and 82 after commencing the series of injections offered the possibility of following the development of pathological alterations in the motor neurons and also in the whole spinal cord. The IgG accumulation in the cytoplasm was prominent in the ALS serum-injected animals on the fourth day and it remained strong throughout the study (Figure 8A,E) compared to the slight immunoreactivity in the motor neurons of the control serum inoculated animals. (Figure 8B). On experimental day 4, small drops in the cytoplasm appeared, large inclusion-like nuggets could be seen on day 21 (Figure 8E), and the whole cytoplasm was immunostained for IgG on day 82 (Figure 8A). The nucleus always remained free of immunostaining for IgG. There was no accumulation of IgG in the dorsal horn neurons and no morphological alteration developed in them. The corticospinal tract, located in the posterior columns of the spinal cord in mice, was also negative for IgG staining. Neuronophagia was noted only in ALS serum-injected animals (Figure 9A,B). The activation of microglia (an increase in the size and positioning in the vicinity of the motor neurons) was continuously noted throughout the experiment in the ventral horns of ALS serum-injected animals (Figure 8C,F). A similar activation of the microglia was not observed in the ventral horn of the spinal cord of mice inoculated with normal human serum (Figure 8D). Astrocytosis (more GFAP immunoreactive cells with enlarged processes) was also seen in the ventral horn of ALS serum-injected animals (Figure 9C), but this was not obvious compared to controls (Figure 9D). The quantitative analysis revealed that in ALS serum treated mice, in the ventral horns of the lumbar spinal cord, the GFAP-positive relative area fraction raised to 20.61% ± 1.51% compared with the 13.66% ± 0.22% control value (*p* = 0.0012; Student t-test). The IBA-1-positive area fraction in the ALS-serum treated mice was also significantly higher (14.16% ± 1.12%) in the ventral horns compared with the controls (9.54% ± 0.76%; *p* = 0.0084, Student t-test).

In summary, the chronic ip administration of ALS serum in mice causes an increased accumulation of IgG and increased intracellular calcium in the spinal motor neurons, microglia activation and slight astrocytosis in the ventral horn of the spinal cord, a significant loss of the spinal motor neurons, increasing weakness and severe degeneration in the perikaryon of the motor neurons and degradation of the motor axon terminals at the neuromuscular junctions.

## 3. Discussion

We observed gradually developing weakness in mice inoculated with the sera of ALS patients and a massive loss of motor neurons in histological sections from their spinal cords. The sera were administered ip, but their IgG content accumulated in spinal motor neurons. The route for the IgG could only be the absorption into the blood, uptake in the axon terminals and transport retrogradely to the perikarya of spinal motor neurons because we demonstrated it in our short-term passive transfer experiment [13]. However, after the 2-day inoculations, neither weakness nor motor neuron loss was noted. Only the early ultrastructural signs of the degeneration developed that time, i.e., the dilation of the cisterns of the Golgi system and the rough endoplasmic reticulum, mild destruction of mitochondria with increased accumulation of calcium in these ultrastructural organelles. The effect of purified IgG on motoneuronal calcium accumulation was demonstrated in other laboratories, as well [22,23], furthermore various specific effects of ALS IgG were described related to other cell types playing a role in the pathomechanism of ALS. ALS IgG was shown to induce oxidative stress and upregulation of the antioxidative system in a BV-2 microglial cell line [24]. It was also shown to affect cytosolic Ca-homeostasis in cultured rat astrocytes [25]. Furthermore, ALS IgG induced selective motor neuron apoptosis in rat mixed primary spinal cord cultures [26]. Nevertheless, during the long-term administration of IgG-containing serum, a severe destruction of the motor axon terminals and a significant loss of motor neurons was observed. The remaining motor neuron perikarya were also severely injured. Furthermore, the animals became gradually weaker. Otherwise, with ALS serum we could transfer most of the signs of the motor neuron disease. With the long-term administration of the serum, its IgG content exaggerated the signs of the acute passive transfer and this led to the death of a significant number of spinal motor neurons in 82 days. As we perfused the animals one week after the last injections on day 82, we were able to exclude the signs of an acute effect of the last injection on the motor neurons. Permanent damage developed, which was observed one week after the discontinuation of the treatment. The gradual loss of motor neurons also reinforced this hypothesis. It is likely that the ALS IgG exerts similar effect on motor neurons of the patients. As we reported, high levels of IgG antibody were detected in the patients’ sera directed to different intracellular proteins that have crucial functions [18]. The antibodies can alter their functions by binding to them. In addition to this, the antibodies constitute inclusions similar to the altered proteins in genetic forms of ALS. As was demonstrated, these high levels of IgG antibodies were specific for the patients, differentiated them from controls [18], thus their ALS-specific effect might be certainly assumed. Since all the patients participating in the study were newly diagnosed, and had not been treated yet when their sera were taken, it is unlikely that some drugs due to the medication is responsible for the severe damage of motor neurons in the recipient mice. As there is no evidence about the etiological role of autoimmunity in ALS, it cannot be decided why and how IgG directing to proteins of motor neurons are produced in ALS. Motor neurons die during aging like other neurons. The immune system of certain individuals can be sensitized to their proteins and can accelerate the otherwise slow normal process. Alternatively, motor neurons can degenerate because of many reasons (see the introduction), hence the release of their protein content can trigger the activity of the immune system to further exaggerate their damage. In any case, IgG mediated processes should be considered as part of the mechanism of ALS, which complicate the progression of the disease.

In the present passive transfer experiment, both the cell bodies and the axon terminals seem to be severely damaged. The calcium content is also increased both in the axon terminals and in the perikarya. The destruction of the mitochondria is prominent in both sites. The source of the increase of calcium is not fully understood. An effect of ALS IgG on L-type calcium channels was suspected [12]. Although electrophysiological studies support this view [27,28], other studies cast some doubt on this idea [29].

Nevertheless, a high level of antibodies directed to Mucolipin 3 was found in the sera of ALS patients [18]. This protein is a novel calcium channel, which releases calcium from endosomes and lysosomes. An increased release of glutamate from the synaptic boutons might also be the source of increased intracellular calcium acting on glutamate receptors [30]. As the rough endoplasmic reticulum is a significant calcium store in the cells, antibodies directed to it might release calcium into the cytosol. As for the lack of the external membrane of the axon terminal (Figure 6B) caused by damage from the passive transfer of the serum of ALS patients, its extensive destruction may also be the source of the extreme influx of extracellular calcium. Whatever the mechanism of the increase in intracellular calcium is, it may take center stage in the pathomechanism of the motor neuron degeneration [31]. The fact that a similar increase of calcium was found in the neuromuscular junctions obtained from biopsy samples of ALS patients [11] further reinforces this idea. However, in our experimental paradigms, the initial step is the effect of anti-motoneuronal IgG. The notion of the mechanism of cell death initiated by multiple intracellular antibodies binding to proteins which have crucial functions in the maintenance of the integrity of the functions of the motor neurons is a novel one.

Knowing that similar proteins can be found in many other neurons, why do only motor neurons die in the CNS? One reason is that only motor neurons have axon terminals outside the blood-brain barrier, and they can take up the IgG antibodies. Microtubules can then transfer the antibodies to the perikarya. The other reason is that the motor neurons contain just a small amount of calcium binding proteins (parvalbumin and calbindin-D28k), and hence, they are unable to protect themselves from the calcium load [32,33].

While by day 4, there was no decrease in the number of motor neurons in the ventral horns of ALS serum-treated mice compared to the controls, three weeks later, only 60% of the motor neurons survived, which further decreased to 42% by the end of the experiment on day 82. At this time, the attributes of ultrastructural damage and the intracellular alterations of calcium in the motor neurons that survived were similar to those observed in the acute experiment performed earlier [12], but the magnitude of the damage was greater. Nevertheless, the animals could move in their cages, and eat and drink, but their isometric muscle strength was diminished, as assessed with the hanging test. The length of the time that they were able to hang gradually decreased to about one third of that of the control group. Based on the above data, the loss of motor neurons was faster in the first three weeks than it was later in the 82-day period. One possible reason for the decreasing rate of neuronal loss might be that over the course of the experiment, the animals started to produce neutralizing antibodies directed to the inoculated anti-motor neuron IgG. Over the course of the experiments, the hanging time also slowly decreased in the control animals, but the decrease was significantly smaller than in the animals injected with the serum taken from ALS patients. When the injections were stopped, the control animals regained their original strength, as indicated by similar hanging times to those measured at the beginning of the experiments. In contrast, the group of ALS serum-injected mice exhibited no recovery of muscle strength. Here, we hypothesize that the frequent intraperitoneal injections through the abdominal wall weakened the abdominal muscles temporarily in both groups. This would then explain the temporary and slight decrease in the muscle strength in the group of control animals.

Our results indicate that a low number of motor neurons is sufficient for the horizontal movement of the animals, even if the neurons are showing the signs of degeneration. This accords with the observation that the onset of symptoms in ALS patients seems to occur after the majority of motor neurons have already been lost [34].

In our previous short-term passive transfer model, the acute administration of ALS IgG and anti-motoneuronal IgG induced microglia recruitment after 2 days of ip administration [15]. Hence, we were interested to see what would happen in the case of chronic administration of ALS sera. We observed the morphological signs of the activation of microglia with the increase in size, having more processes and positioning close to the motor neurons (satellitosis), which was demonstrated on the fourth day of the experiment and it was regularly observed up to the last perfusion. Astrogliosis in the ventral horn seemed to increase by the end of the experiment. The exact role of glial cells in the pathomechanism of ALS is still unclear. Dysregulation of inflammatory pathway is generally present in ALS patients [35], and also characteristic of animal models [36], which was replicated in the present study, as well. As their role is considered, microglia [37] and astrocytes [38] may have an either beneficial or detrimental role in the pathomechanism of ALS. Feasibly, they can be considered as double-edged swords, providing neuroprotection at the early phase of the disease, but may trigger irreversible pathological processes at a later phase, resulting in non-cell autonomous death of motor neurons [39].

We think that the accumulation of IgG in the motor neurons can form aggregations that bind to their targets. However, studies conducted in various transgenic animal models for ALS reveal that symptoms and the loss of motor neurons do not always correlate with the appearance of neuronal inclusion bodies [40]. Nonetheless, based on our observations, we may conclude that morphological signs of neuronal degeneration and loss are not just associated with different inclusions containing genetically pathological protein aggregates. Inclusions made of intracellular IgG shown to be bound to intracellular micro-organelles may be sufficient to cause cell death by impairing or inhibiting the normal functions of motor neurons. It suggests that there is a unique mechanism of cell death, especially for neurons having projections outside of the blood-brain barrier, hence it is able to uptake IgG directed specifically to their internal structural antigens. In our previous acute passive transfer experiments, one or two days after the appearance of ALS IgG in the lower motor neurons, activated microglia cells were recruited to the vicinity [15], elevated levels of proinflammatory cytokines TNF-α and Interleukin 6 were found in the spinal cords, and later in the blood too. [17]. Even though these observations may explain a possible mechanism of death of motor neurons, in these acute experiments neither the loss of motor neurons nor weakness of the inoculated mice was observed. In the present long-term passive transfer experiment, the serum (probably with the anti-motoneuron IgG and cytokines) is sufficient to initiate a process, and this eventually leads to the death of motor neurons.

## 4. Materials and Methods

### 4.1. Patients

Blood samples were taken from 25 ALS patients (15 women, 10 men) and 10 age-matched normal controls (6 women, 4 men). The patients should be regarded as sporadic ALS patients because the disease did not occur in their families. Nevertheless, all the ALS patient’s DNA was screened for 35 major ALS related genes with known mutations posteriorly. A *superoxide dismutase-1* (*SOD1*) mutation in the DNA of a woman, a *never in mitosis gene a-related kinase 1* (*NEK1*) mutation in a man and an *ALS2* mutation in another man were detected. However, the family history of all three patients was negative for signs of any neurodegenerative disease, including ALS. A genetic analysis of ALS patients was performed using genomic DNA isolated from frozen blood. Initially, all patients were screened for mutations in the most frequently mutated genes in ALS; *chromosome 9 open reading frame 72* (*C9orf72*) and *SOD1* [41]. Patients who did not carry pathogenic variants in *C9orf72* or *SOD1* genes, were subjected to targeted next-generation sequencing (NGS). The ALS related gene panel used for targeted NGS was designed using SureDesign (Agilent Technologies) covering the coding regions of 35 ALS related genes: *ANG* (*Angiogenin*), *ALS2*, *ATXN2* (*Ataxin 2*), *C9orf72*, *CCNF* (*G2/mitotic-specific cyclin F*), *CHCHD10* (*coiled-coil-helix-coiled-coil-helix domain containing 10*), *CHMP2B* (*charged multivesicular body protein 2B*), *DAO* (*D-amino acid oxidase*), *DCTN1* (*dynactin-1*), *ELP3* (*elongator complex protein 3*), *ERBB4* (*transmembrane tyrosine kinase receptor*), *EWSR1* (*Ewing sarcoma break point region 1*), *FIG4* (*phosphoinositide 5 phosphatase*), *FUS* (*fused in sarcoma*), *GLE1* (*nucleoporin GLE 1 protein gene*), *GRN* (*granulin gene*), *HNRNPA1* (*heterogeneous nuclear ribonucleoprotein A1*), *MATR3* (*Matrin 3*), *NEFH* (*neurofilament heavy*), *NEK1*, *OPTN* (*optineurin*), *PFN1* (*profilin 1*), *SETX* (*senataxin*), *SIGMAR1* (*sigma-non-opioid intracellular receptor 1*), *SOD1*, *SPG11* (*spataxin*), *SQSTM1* (*sequestosome 1*), *SS18L1* (*NBAF chromatin remodeling complex subunit*), *TAF15* (*TATA-Box binding protein associated Factor 15*), *TARDBP* (*transactive response DNA-binding protein gene*), *TBK1* (*TANK binding kinase 1 / NF-Kappa-B-activating kinase*), *TUBA4A* (*tubulin α 4A*), *UBQLN2* (*ubiquilin 2*), *VAPB* (*vesicle-associated membrane protein gene*) and *VCP* (*valosin containing protein gene*). Sequencing was performed on the Illumina NextSeq 500 sequencer (Illumina Inc. San Diego, CA, USA). The bioinformatic analysis of sequencing data was then performed to identify single nucleotide variants and small insertions/deletions according to the best practices of Genome Analysis Toolkit (GATK) [42].

The average age of the patients was 60 ± 10.74 (s.d.) years, while the average age of the controls was 59.8 ± 10.19 years. The patients were consecutively examined at the Department of Neurology, University of Szeged. The controls were volunteers, disease free, and age-matched to ALS patients. Each individual in this study gave their informed consent for the use of their sera in this experiment. We used the sera taken from normal individuals as controls. The reason for this was that although in our previous experiments IgG from patients with degenerative or autoimmune neurological diseases caused no ultrastructural alterations in the perikaryon in mice, it did cause slight alterations of the calcium homeostasis in the axon terminals. IgG from myasthenia gravis and Guillain-Barré syndrome increased, while IgG from Eaton-Lambert myasthenic syndrome and multifocal motor neuropathy decreased the intraterminal calcium [12]. The diagnoses fulfilled the El Escorial revisited [43] and the Awaji [44] criteria for ALS). The patients were followed up several times at our institution and the progression was recorded with the revised ALS functional rating scale (ALSFRS-R) [45]. However, all of the patients had already died at the time of the experiment. Six of them underwent autopsy which confirmed our diagnosis. The blood samples and after centrifugation of the sera were kept at −80 °C until use.

### 4.2. Ethics Approval and Consent to Participate

Ethical approvals of the studies involving animals were given by (1) The Government Office in Csongrád County, Hungary; project title: Therapeutical Perspectives of Neurological Diseases—Research for Biomarkers in Neurodegenerative Diseases. #XI/4962/2015 [2015.01.14]. (2) Committee for Animal Experiments of the University of Szeged, Szeged, Hungary I. 74-II/2015 [2015.01.14]. (3) All the experiments were carried out in accordance with the institutional guidelines for the use and care of the experimental animals and governmental law for animal protection. The experimental protocols and the animal care were approved by the Ethical Committee for the Protection of Animals in Scientific Research at the Biological Research Center of the Hungarian Academy of Sciences (approval No. 72-45/b/2001 [2001.09.01] and No. 03876/0014/2006 [2006.10.28]) and carried out in accordance with the national law (XXVIII. Chapter IV. paragraph 31) which conforms to international laws and policies (EEC Council Directive 86/609, OJL 358 1 DEC. 12, 1987; NIH Guide for the Care and Use of Laboratory Animals, United States National Research Council, revised 1996). Every effort was made to minimize animal suffering throughout the experiments. The ethical approvals for obtaining blood from patients and controls, using it for research purposes, storing it in an anonymous manner with the written informed consent of patients and controls were given by The Human Investigation Review Board, University of Szeged, Hungary. Project title: Search for Biomarkers in Neurodegenerative Diseases: Amyotrophic Lateral Sclerosis, Parkinson Disease and Alzheimer Disease #2557/2009 [2009.06.29, revised 2012.01.23]. They stated that the project agreed with the declaration of the Medical World Federation proclaimed in Helsinki 1964.

### 4.3. Ip Inoculation of Mice with ALS and Control Sera, Measuring Muscle Strength and Perfusion for Histological Examination

At the beginning of the experiment, 32 male and female mice were in the group which were given 0.3 mL/day of serum taken from ALS patients ip. On the same days 16 mice (both males and females) were given the same amount of control sera. Next, the inoculations were continued every other day. At a given injection time the whole group of ALS serum injected animals were given serum from one patient and the control serum injected animals were given serum from one normal individual. On the next injection day, the groups were given serum from another ALS patient or from another normal control individual and so on. That is to say, one group of animals received only different ALS sera, the other only different normal control sera.

On the days of measuring muscle strength and one day before such tests, inoculations were not performed. In total, during the experiment, the animals in the longest survival period were injected 35 times. Their average weight was 22.32 ± 2.18 g. The source of the Balb/c mice was Charles River Appoints AnimaLab Hungary Kft. (Vác, Hungary).

The animals were housed in plastic cages (at most 5 animals/cage) in a thermoneutral environment (21 ± 3 °C) and in a 12 h light/dark cycle with access to drinking water and regular rodent chow ad libitum.

Altogether, 3 animals receiving ALS serum died between two injections at night and one during the anesthesia before inoculation at different time points (7th, 10th, 27th injection). The preceding two days their gait became sluggish. Because of these unexpected events they were excluded from the statistical analysis. However, a mouse receiving 26 injections was devoted to routine histologic examination, which proved significant loss of motor neurons. To determine the baseline level of the isometric force of the animals before commencing the series of ip inoculations of the sera, we measured the time that the animals were able to maintain themselves hanging with four legs upside down on the bars of their cage. We choose 30 min as the upper limit of hanging in the experiment, although most of the animals were able to hang longer than 50 min during tests before the experiment. The aim this was to set this limit so as to avoid the exhaustion of the animals. To characterize the loss of muscle strength during the experiments, we put the mice on the inverted cage upside down and measured the time elapsed between the beginning of hanging to dropping down to a smooth, cushioned surface placed 50 cm under their position. After becoming weaker, they dropped from the bars sooner (video Supplementary Materials). This test is based on the knowledge that mice remain hanging on the grid till exhaustion; it is easy to perform, reliable and reproducible [46] and, in addition, it does not require a pre-test teaching period. The assay as implemented in our laboratory measures the conditions of the four limbs (see video Supplementary Materials), furthermore, since it is not detrimental, it can be used in a longitudinal manner [47]. The hanging times were measured on days 4, 11, 21, 40, 68, 76 and 82 of the experiment.

On the fourth day of the experiment, two controls and two ALS injected males and two females were given inhalation anesthesia with Flurane, (Biocare Pharmaceutica, Lahore, Pakistan), then perfused through the heart with phosphate buffered saline (PBS) and 4% paraformaldehyde, 0.05% glutaraldehyde and 0.2% picric acid containing fixative (suitable for electron microscopic examination and also for immunohistochemistry). One control, 1 male and 1 female ALS serum injected animal was perfused with only 4% paraformaldehyde in PBS for histological examination. Altogether, 5 animals died during the anesthesia before inoculation at different time points; hence, they were excluded from the statistical analysis.

On experimental day 21, 6 controls and 8 ALS serum injected animals were perfused under general anesthesia with phosphate buffered saline and fixed with perfusion for histological and histochemical examinations (three controls and 4 ALS serum treated animals were perfused with glutaraldehyde and picric acid containing 4% paraformaldehyde, the others with 4% paraformaldehyde. Seven days after the discontinuation of the injections on experimental day 82, 4 controls and 8 ALS serum-injected mice were perfused for histological examination as above. Three controls and 5 ALS serum-injected animals were then perfused for an electron microscopic examination and ultrastructural calcium imaging.

### 4.4. Histological Processing of the Spinal Cords for Light Microscopic Examination

The spinal cords were removed from the perfused animals and kept in the fixative for 3 days at 4 °C. Then they were embedded in paraffin and 3 µm thick cross sections were cut with a microtome and put on Menzel-Glaser Superfrost Ultra Plus glass slides (Thermo Scientific; Braunschweig, Germany). The sections were deparaffinized in xylene, rehydrated in a graded series of ethanol and stained with hematoxylin-eosin (Sigma Chemical Company; St. Louis, MO, USA), or were processed for immunostaining. The details are shown in Table 1.

Two pretreatment methods were applied (abbreviated as TRS high or low in the table). TRS low refers to citrate buffer used at pH6.1 (K8005 Target Retrieval Solution, Low pH; Agilent Technologies/Dako, Santa Clara, United States) for 20 min at 95 °C in a Dako PT Module. In contrast TRS high can be specified as a Tris/EDTA pH9 buffer (K8004 target retrieval solution high pH; Agilent Technologies/Dako, Santa Clara, United States) for 20 min at 95 °C in a Dako PT Module. Moreover, 1 min concentrated formic acid (F.A.) was used when needed. After immunostaining, sections were counterstained with Mayer’s hemalum solution (Merck #109249) for 30 s, differentiated in 0.45% HCl in 70% ethanol for a few seconds, rinsed in warm tap water for bluing, dehydrated in graded ethanol, cleared in n-butyl acetate and coverslipped with Consul-Mount (Thermo Scientific™ Shandon™, Ref# 9990440). As a control, the primary antibodies were left out of the process and no specific immunostaining was noted. GFAP: glial fibrillary acidic protein, a marker for astrocytes. IBA-1: ionized calcium-binding adapter molecule 1: microglia marker. The sections were examined under a Zeiss Axio light microscope, photographed with the attached Axio-Cam MRc and the images were saved in the Axio-Vision program on a computer.

Selected sections obtained from the lumbar segments of the spinal cord of mice were used for the quantification of the extent of astrocytosis (GFAP immunostaining) and microgliosis (IBA–1 immunostaining). Mice having been injected either with ALS serum (*n* = 8) or with control serum (*n* = 4) (Table 2) were sacrificed on experimental day 82. 7 sections from control- and 10 sections from ALS-serum injected animals were used for quantification of GFAP staining. 9 sections from control- and 16 sections from ALS-serum treated animals were used for measuring the extent of IBA-1 staining. The views of ventral horns from the lumbar spinal cords were examined in a Zeiss Axio light microscope, photographed with the attached Axio-Cam MRc and the images were saved in the Axio-Vision program in tagged image file format. The recorded images were analyzed with the built-in modules of the Image-Pro Plus (Media Cybernetics; Rockville, MD, USA) image analysis software supplemented with a macro module which was developed in our laboratory. Then we expressed the percentage of the significantly stained profiles relative to the total area of the field of views [48].

### 4.5. Counting the Motor Neurons in the Spinal Cord of Animals

To reduce the number of animals used in the study, no groups were formed intended solely for counting motor neurons. Instead, at the intermediate time points, some of the histologic sections prepared for immunohistochemistry were allocated for cell counting using the optical disector method [49]. Alternatively, at the end of the experiment, some of the plastic sections prepared for electron microscopic calcium analysis were selected for cell counting using the physical disector method. [50].

For histological characterizations, the lumbar spinal cord was chosen, because in all of the immune-mediated animal models of motor neuron destruction and some other models (e.g., SOD-1 mutant transgenic mice) hind limb weakness develops as a first sign of the disease. Motor neurons were counted light microscopically in a set of the equidistantly (at 108 µm) placed sections, selected from the collection of consecutively cut sections from the lumbar intumescence of the spinal cords of each animal. The sections meant just for cell counting were stained with hematoxylin-eosin. The motor neurons were identified by their size, their location in the ventral horns and by their morphological features. To satisfy the selection criterion of the disector method, i.e., to identify the object to be counted with its single, point-like structure, motor neurons with discernible nucleolus were counted. As summarized in the Table 2, at day 4, 3 control- and 6 ALS serum injected mice, at day 21, 6 control- and 8 ALS serum injected mice, and at day 82, 4 control- and 8 ALS mice were used for counting motor neurons in the histologic sections of the lumbar segment of the spinal cords. At each time points, 23 sections were screened for the presence of motor neurons, in average. The sections were examined under a Zeiss Axio light microscope, photographed with the attached Axio-Cam MRc and the images were saved in the Axio-Vision program on a computer. Motor neuronal count were pooled for animals and expressed as a volume density (number/mm^3^), according to the total examined volume (total area examined multiplied by a 3 µm section thickness) of the spinal cord in each animal. The average number of motor neurons in the lumbar segments was determined for each treated group at each time point.

At the end of the experiment, on day 82, using sets of pairs of plastic sections prepared for electron microscopic calcium analysis, an unbiased estimation of the number of motor neurons in the cervical and lumbar segments of the spinal cords was performed using the physical disector method. As summarized in Table 2, 3 control serum treated and 5 ALS serum treated mice were used for the analysis. From each animal 12-12 dissectors were prepared from the cervical and the lumbar segments of the spinal cord of each animal. The purpose of extending the analysis to the cervical segments was to determine whether the changes in the number of the motor neurons were homogeneous across the spinal cord. Pairs of semi-thin sections with a thickness of 0.5 µm, cut at distances of 8 µm, were etched to remove the resin, stained and used as reference- and look-up planes for calculations. The nominal distance between each individual disector was 40 µm, an interval necessary to ensure we did not count the same cells twice. The motor neurons were identified by their size, location in the ventrolateral position and their ultrastructural features. The anatomical border of the ventrolateral pool of motor neurons was determined using the method described by Kong [51]. The cells were counted if they met the selection (the point-like identification) criterion of the disector, which in this case was the presence of the nucleus in the reference section and absence of the same nucleus on the look-up plane. To match tissue regions on the reference and look-up sections, as well as to apply the counting rule of the disector, corresponding image pairs were recorded with a MicroPublisher 5.0 RTV charge-coupled device camera (QImaging; Surrey, Canada) attached to an Eclipse 80i (Nikon) light microscope, then examined with the Image-Pro Plus image analysis program and disector counts were pooled for animals, and the average number of motor neurons in the cervical and lumbar segments was determined for each group.

### 4.6. Electron Microscopic Fixation and Detection of Calcium in Spinal Motor Neurons

The mice were anesthetized with Avertin (tribromoethanol, Fluka; Buchs, Switzerland; 240 mg/kg body weight in 1 mL volume intraperitoneally), then they were perfused transcardially with 90 mM potassium oxalate (Sigma-Aldrich, St. Louis MO, USA) adjusted to pH 7.4 with KOH and followed by 3% glutaraldehyde, also adjusted to pH 7.4 using KOH (Polysciences; Warrington, PA, USA). The method which was originally described by Borgers and colleagues to demonstrate the distribution of tissue calcium on the electron microscopic scale [52,53], and was adapted, and regularly used in our laboratory [54,55,56,57], results in good structural preservation of the tissue, and electron dense deposits where the tissue calcium is precipitates by the fixative. In our earlier studies, the applicability of the technique was proved in a wide variety of experimental paradigms to detect slowly evolving function-dependent changes of total intracellular calcium, including acute [58] or chronic oxidative stress [59], acute ischemia [60] or after axotomy [61,62]. In these experiments, as well as during the present study the specificity of the reaction for calcium was regularly checked by means of energy dispersive X-ray microanalysis or electron spectroscopic imaging (see e.g., [31] or [63]). The preparation method, as stated briefly, after perfusion, the cervical and lumbar segments of the spinal cords and the interosseus muscles in the fore limbs and the hind limbs were removed and postfixed in the same fixative overnight at 4 °C. Afterwards the specimens were rinsed in a mixture of 7.5% sucrose (Molar; Halásztelek, Hungary) and 90 mM potassium oxalate (pH 7.4), postfixed with 2% pyroantimonate (Merck; Kenilworth, NJ, USA) containing 1% osmic acid (Sigma; adjusted to pH 7.4 with acetic acid (Molar) for 2 h at 4 °C. Then the specimens were rinsed in distilled water (pH 10, adjusted with KOH) for 10 min, dehydrated in a graded series of ethanol (Molar), processed with propylene oxide (Sigma) and embedded in Durcupan ACM (Fluka Sigma Aldrich, St. Louis MO, USA). The blocks were polymerized at 56 °C for two days. Then 0.5 µm semithin sections were cut from the blocks on an Ultracut UCT ultramicrotome (Leica; Wetzlar, Germany) etched [64] and stained according to Richardson’s procedure [65]. The sections were evaluated under an Eclipse 80i microscope (Nikon; Tokyo, Japan) to identify pools of motor neurons in the ventral horns of the spinal cords and the motor axon terminals at the neuromuscular junctions of the interosseous muscles. After trimming the blocks to the appropriate regions, systematic sets of ultrathin sections (40 nm) were cut, mounted on formvar coated single hole copper grids (Electron Microscopy Sciences; Hatfield, PA, USA) and contrasted with 2% uranyl acetate (Electron Microscopy Sciences) in 50% ethanol and 2% lead citrate (Electron Microscopy Sciences) in distilled water. The distance between the sections was 15 µm and 40 µm in muscle samples and in spinal cord samples, respectively, in order to avoid sampling identical motor axon terminals or motor neurons during the evaluation of electron microscopic fields of views.

### 4.7. Quantification of Intracellular Calcium and Mitochondrial Volume in Motor Neurons

The ultrathin sections described above were analyzed in a JEM-1400Flash transmission electron microscope (JEOL; Tokyo, Japan). The sections were systematically screened at low magnification (1000–3000×) for the presence of the profiles of motor neurons in the ventral horns of the cervical and the lumbar spinal cord. Screening was identical for the motor axon terminals in the interosseous muscles. The sampling was continued until 15 fields of view had been analyzed from each muscle and spinal cord area of each animal. The relative volume of the perikarya and the partial volumes of the axon terminals occupied by the EDDs representing the calcium precipitates were determined by point counting methods [66,67], which were modified for these unique structures and photographic conditions [60,68,69]. Briefly, 8-bit grayscale images were recorded at an instrumental magnification of 12,000× (for axon terminals) or at 20,000× (for motor neuron somas) with an 8 MP charge-coupled device camera (JEOL) and saved in tagged image file format. The recorded pictures were analyzed with the built-in modules of the Image-Pro Plus (Media Cybernetics; Rockville, MD, USA) image analysis software. Tessellation of sampling points was superimposed on each electron microscopic image, then sampling points matching the perikaryal profile or the axon terminals in each image served as reference areas and were counted. Sampling points matching the EDDs within the reference area were counted as well. The corresponding counts obtained in the individual fields were summed up throughout the series of the identified motor neuron perikarya or axon terminals in each animal. The appropriate ratios that expressed the relative amount of EDDs within these structures were then calculated for each animal. The partial volume of mitochondria in the motor axon terminal and in the perikaryal space was determined at day 82. For this analysis, images were recorded and stored and analyzed as above. Tessellation of sampling points was superimposed on each electron microscopic image similarly, then sampling points matching the perikaryal profile or the axon terminals in each image served as reference areas and were counted. Sampling points matching the mitochondrial profiles within the reference area were counted as well. The corresponding counts obtained in the individual fields were summed up throughout the series of the identified motor neuron perikarya or axon terminals in each animal. The appropriate ratios that expressed the relative mitochondrial volume within these structures were then calculated for each animal. Perikaryal data obtained from the cervical and lumbar motor neurons, as well as axon terminal data obtained from the hind limb and fore limb interosseous muscles were pooled.

### 4.8. Statistical Analysis of the Data

To determine the average volume occupied by EDDs within the cellular compartments of motor neurons, the data values derived from individual electron microscopic fields were pooled in terms of the animals, neuronal regions and passive transfer groups. Fifteen fields of view were analyzed in the perikarya taken from each animal, with three animals in the control serum-injected group and five animals in the ALS serum-injected group. In the case of passive transfer with ALS serum, sexual dimorphism was tested with Student’s t-test, but male and female groups did not display significant difference (*p* = 0.98), so the data values were combined. In the axon terminals found in the interosseous muscles fifteen fields were obtained from each animal. Differences among multiple means of the volume density of the EDDs were assessed by a one-way analysis of variance (ANOVA), followed by a least significant difference post-hoc test. Student’s t-test was applied to evaluate the differences between the groups at each time point for the muscle strength measurements and counting the number of motor neurons, mitochondrial partial volumes and relative area fraction occupied by microglia or astrocytes. In a similar way, the statistical significance of the difference between the neuronal survivals was determined via Student’s t test. All of the statistical analyses were then performed with R (version 3.0.2) with R Studio Integrated Development Environment (version 3.0) for Windows. All the data values are presented as mean values ± the standard error of the means (s.e.m.).

## 5. Conclusions

In our quest to learn more about the pathomechanism of ALS, we decided to transfer the symptoms to mice by using sera taken form ALS patients. Serial ip inoculations of the sera of ALS patients in mice caused spinal motor neuron loss and weakness of the animals. IgG from the serum was taken up in motor neurons and it accumulated in the cytoplasm. At the same time, intracellular calcium rose markedly in the axon terminals and in the perikarya. Signs of ultrastructural damage were observed in the neurons after one week of the discontinuation of the serum injections; this seemed to be most advanced in the axon terminals at the neuromuscular junctions. Our experiment demonstrated that the chronic ip administration of serum of ALS patients is sufficient to induce motor neuron death in mice, leading to more than 40% cell loss as early as the 21st day of the treatment which, along with the documented muscle weakness, can be considered as a middle stage disease in mice modeling ALS [36]. Regarding this and our previous experiments, we think that the continuous uptake and binding of anti-motoneuron IgG to functionally important proteins can seriously alter the functions of the whole cell. The increase in intracellular calcium in the acute and also in the chronic treatment with the serum or IgG taken from ALS patients may play a central and self-perpetuating role in the pathomechanism of cell death. However, the relationship between the effect of antibodies and the increase in intracellular calcium is, at present, not completely clear.

## Figures and Tables

**Table 1:** Methods for immunostaining of spinal cord sections.

Antigen	Pre-Treatment	Blocking	Primary Antibody	Visualization System
IgG (human)	TRS low	**M.O.M. block:** 1h (Vector #MKB-2213) **H2O2 Block:** Flex+ K8002 5min	rabbit polyclonal **1/4000** 30min Dako #A0423	DakoAutostainer 48 Link platform: DakoEnVision FLEX+ (#K8002, Agilent Technologies/Dako, Santa Clara, United States) used according to manufacturer’s instructions with rabbit linker with DAB as chromogen
IBA-1	TRS high	**H2O2 Block:** 0,9% H2O2 in methanol for 10min **M.O.M. block:** 1h (Vector #MKB-2213) **Proteinblock:** 1h 10% FCS in Tris	rabbit polyclonal **1/2000** overnight Wako (Osaka, Japan) #019-19741	manual staining: Shandon Coverplates and Sequenza Immunostaining racks (Thermo Scientific #72110017) Detection:Dako REAL™ EnVision™ Detection System (#K5007, Agilent Technologies/Dako, Santa Clara, United States) used according to manufacturer’s instructions with DAB as chromogen
GFAP	TRS low	**M.O.M. block:** 1h (Vector #MKB-2213) H2O2 Block: Flex+ K8002 5min	rabbit polyclonal **1/1000** 30min Dako #Z0334	DakoAutostainer 48 Link platform: DakoEnVision FLEX+ (#K8002, Agilent Technologies/Dako, Santa Clara, United States) used according to manufacturer’s instructions with rabbit linker with DAB as chromogen

**Table 2:** The number of animals allocated to different tests at different survival times.

Survival Time	Method	Control Serum Treated	ALS Serum Treated
**day 4**	hanging test cell counting and histology	*n* = 16 *n* = 3	*n* = 32 *n* = 6
**day 11**	hanging test	*n* = 13	*n* = 26
**day 21**	hanging test cell counting and histology	*n* = 13 *n* = 6	*n* = 23 *n* = 8
**day 40**	hanging test	*n* = 7	*n* = 14
**day 68**	hanging test	*n* = 7	*n* = 13
**day 75**	hanging test	*n* = 7	*n* = 12
**day 82**	hanging test cell counting and histology stereology and electron microscopy	*n* = 7 *n* = 4 *n* = 3	*n* = 11 *n* = 8 *n* = 5

## Supplementary Materials

Supplementary materials for representing hanging in a video format can be found at https://www.mdpi.com/1422-0067/20/10/2573/s1.

## Abbreviations

ALS	amyotrophic lateral sclerosis
ALSFRS-R	revised ALS functional rating scale
ANG	angiogenin
ANOVA	analysis of variance
ATXN2	ataxin 2
C9orf72	chromosome 9 open reading frame 72
CCNF	G2/mitotic-specific cyclin F
CHCHD10	coiled-coil-helix-coiled-coil-helix domain containing 10
CHMP2B	charged multivesicular body protein 2B
DAO	D-amino acid oxidase
DCTN1	dynactin-1
DNA	deoxyribonucleic acid
EDDs	electron dense deposits
ELP3	elongator complex protein 3
ERBB4	transmembrane tyrosine kinase receptor
EWSR1	Ewing sarcoma break point region 1
FIG4	phosphoinositide 5 phosphatase
FUS	fused in sarcoma
GATK	genome analysis tool kit
GFAP	glial fibrillary acidic protein
GLE1	nucleoporin GLE 1 protein
GRN	granulin
HNRNPA1	heterogeneous nuclear ribonucleoprotein A1
IBA-1	ionized calcium-binding adapter molecule 1
IgG	immunoglobulin G
ip	intraperitoneal
MATR3	Matrin 3
MCP-1	macrophage chemoattractant protein 1
NEFH	neurofilament heavy
NEK1	never in mitosis gene a related kinase 1
NGS	next generation sequencing
OPTN	optineurin
PBS	phosphate buffered saline
PFN1	profilin 1
s.e.m.	standard error of means
s.d.	standard deviation
SETX	senataxin
SIGMAR1	sigma-non-opioid intracellular receptor 1
SOD1	superoxide dismutase 1
SPG11	spataxin 11
SQSTM1	sequestosome 1
SS18L1	NBAF chromatin remodeling complex subunit
TAF15	TATA-Box binding protein associated Factor 15
TARDBP	transactive response DNA-binding protein
TBK1	TANK binding kinase 1 / NF-Kappa-B-activating kinase
TNF-α	tumor necrosis factor alpha
TUBA4A	tubulin α 4A
UBQLN2	ubiquilin 2
UPS	ubiquitin-proteasome system
VAPB	vesicle-associated membrane protein
VCP	valosin containing protein

## Appendix A

During the experiments, isometric muscle strength was characterized by the hanging test. The mouse on the right hand side was inoculated with normal human serum and was able to hang 30 min before the recording. The mouse on the left hand side was treated with serum from ALS patient. In this short video, the control mouse hung the whole time of the video (38 s), while the ALS serum inoculated mouse hung 12 s for the first time and 8 s for the second time. This presentation indicates that the ALS serum inoculated mouse has become weaker and he dropped from the bars sooner compared to the control mouse.
